# miRNAs can be generally associated with human pathologies as exemplified for miR-144*

**DOI:** 10.1186/s12916-014-0224-0

**Published:** 2014-12-03

**Authors:** Andreas Keller, Petra Leidinger, Britta Vogel, Christina Backes, Abdou ElSharawy, Valentina Galata, Sabine C Mueller, Sabine Marquart, Michael G Schrauder, Reiner Strick, Andrea Bauer, Jörg Wischhusen, Markus Beier, Jochen Kohlhaas, Hugo A Katus, Jörg Hoheisel, Andre Franke, Benjamin Meder, Eckart Meese

**Affiliations:** Chair for Clinical Bioinformatics, Saarland University, Saarbrücken, Germany; Institute of Human Genetics, Saarland University, Homburg, Germany; Department of Internal Medicine III, University of Heidelberg, Heidelberg, Germany; Institute of Clinical Molecular Biology, Christian-Albrechts-University Kiel, Kiel, Germany; Department of Gynecology and Obstetrics, University Breast Center Franconia, University Hospital Erlangen, Friedrich-Alexander University Erlangen-Nuremberg, Erlangen, Germany; German Cancer Research Center, Heidelberg, Germany; University Hospital Würzburg, Würzburg, Germany; Comprehensive Biomarker Center, Heidelberg, Germany; German Center for Cardiovascular Research - DZHK, Germany, Heidelberg

**Keywords:** Bioinformatics, Biomarker, Microarray, miRNA

## Abstract

**Background:**

miRNA profiles are promising biomarker candidates for a manifold of human pathologies, opening new avenues for diagnosis and prognosis. Beyond studies that describe miRNAs frequently as markers for specific traits, we asked whether a general pattern for miRNAs across many diseases exists.

**Methods:**

We evaluated genome-wide circulating profiles of 1,049 patients suffering from 19 different cancer and non-cancer diseases as well as unaffected controls. The results were validated on 319 individuals using qRT-PCR.

**Results:**

We discovered 34 miRNAs with strong disease association. Among those, we found substantially decreased levels of hsa-miR-144* and hsa-miR-20b with AUC of 0.751 (95% CI: 0.703–0.799), respectively. We also discovered a set of miRNAs, including hsa-miR-155*, as rather stable markers, offering reasonable control miRNAs for future studies. The strong downregulation of hsa-miR-144* and the less variable pattern of hsa-miR-155* has been validated in a cohort of 319 samples in three different centers. Here, breast cancer as an additional disease phenotype not included in the screening phase has been included as the 20^th^ trait.

**Conclusions:**

Our study on 1,368 patients including 1,049 genome-wide miRNA profiles and 319 qRT-PCR validations further underscores the high potential of specific blood-borne miRNA patterns as molecular biomarkers. Importantly, we highlight 34 miRNAs that are generally dysregulated in human pathologies. Although these markers are not specific to certain diseases they may add to the diagnosis in combination with other markers, building a specific signature. Besides these dysregulated miRNAs, we propose a set of constant miRNAs that may be used as control markers.

**Electronic supplementary material:**

The online version of this article (doi:10.1186/s12916-014-0224-0) contains supplementary material, which is available to authorized users.

## Background

In the past decade, non-coding miRNAs have aroused scientists’ interest and their exploration has revolutionized biology. Since the first miRNA was discovered in *Caenorhabditis elegans* in 1993 [1], an increasing number of miRNAs for various species have been reported. Currently, release 20 of the miRBase [2,3] contains 24,521 entries representing hairpin precursor miRNAs, expressing 30,424 mature miRNA products in 206 species. For *Homo sapiens*, more than 2,500 different mature miRNAs are currently included in this database.

The small non-coding miRNAs are known to be involved in crucial biological processes such as proliferation, apoptosis, differentiation, or development [4-6]. More than 50% of all genes in the human genome are known to be miRNA targets and, thus, miRNAs are involved in the regulation of a manifold of metabolic and regulatory pathways such that now the integrative network analysis of miRNAs and mRNAs becomes more and more possible [7-9]. Hence, abnormal miRNA profiles have been associated with many human pathogenic processes as shown by many studies that focused on tissue-derived miRNA profiles (e.g., from patients with lung cancer [10], breast cancer [11], or glioblastoma [12]). Since these small nucleic acids excel in their high stability, they have become even more attractive as biomarker candidates. This also underlines the potential of miRNA biomarkers derived from peripheral blood for diagnostic purposes. Many groups investigated circulating miRNA profiles from serum for various diseases (non-ischemic systolic heart failure [13], pulmonary tuberculosis [14], non-small-cell lung cancer [15,16], breast cancer [17], prostate cancer [18], or ovarian cancer [19]), whereas we and others developed standardized operating procedures for measuring miRNA profiles from whole peripheral blood (myocardial infarction [20], lung cancer [21], multiple sclerosis [22,23], melanoma [24], ovarian cancer [25], chronic obstructive pulmonary disease [26], glioblastoma [27], and Alzheimer disease [28]).

In the present meta-analysis, we analyzed a total of 848 miRNAs in 1,049 samples (containing the 454 samples published in our previous study [29]) measured from whole blood collected in PAXgene blood tubes. The investigated cohort includes healthy controls as well as patients diagnosed with one of 19 diseases of different International Classification of Diseases (ICD)-10 classes (10 cancer entities and 9 non-cancer diseases; details on the different cohort sizes are presented in Table 1). Our results provide a comprehensive overview of the human disease miRNome. By using this rich data source, we aimed at identifying miRNA profiles representative for a general disease state, and to identify miRNA signatures that are suited to discriminate different diseases from controls and from each other.

**Table 1:** **Cohorts with International Classification of Diseases (ICD)-10 code and cohort sizes**

**Disease**	**ICD-10**	**# Samples**	**Institution providing RNA**
**Normal**	–	94	Saarland University
			DKFZ/Heidelberg University
			Heidelberg University
			Julius-Maximilians-University Wuerzburg
			Zürich University
			Christian-Albrechts-University Kiel
**Long-lived individuals**	–	15	Christian-Albrechts-University Kiel
**Tumor of stomach**	C16	13	DKFZ/Heidelberg University
**Colon cancer**	C18	29	Saarland University
**Lung cancer**	C24	73	Saarland University
**Pancreatic ductal adenocarcinoma**	C25	45	DKFZ/Heidelberg University
**Melanoma**	C43	35	Saarland University
**Ovarian cancer**	C56	24	Julius-Maximilians-University Wuerzburg
**Prostate cancer**	C61	65	Saarland University
**Wilms tumor**	C64	124	Saarland University
**Renal cancer**	C65	20	Saarland University
**Glioma**	C71	20	Zürich University
**Sarcoidosis**	D86.0	45	Albrecht Ludwigs University, Freiburg
**Multiple sclerosis**	G35	23	Saarland University
**Acute myocardial infarction**	I21.3	62	Heidelberg University
**Non-ischemic systolic heart failure**	I42	33	Heidelberg University
**Chronic obstructive pulmonary disease**	J40-47	47	Saarland University
**Peridontitis**	K05.4	18	Christian-Albrechts-University Kiel
**Pancreatitis**	K85	37	DKFZ/Heidelberg University
**Psoriasis**	L40	43	Saarland University
**Benign prostate hyperplasia**	N40	35	Saarland University
**Others**	–	149	

## Methods

### Blood samples and groups

The blood samples were collected and processed from nine different institutions (Table 1). Five centers provided samples from individuals with disease as well as controls. Blood was collected in PAXgene Blood RNA tubes (Becton Dickinson). All blood donors participating in this study gave their informed consent and local ethics committees (Ethics Commission at the Friedrich-Alexander University Erlangen-Nürnberg Medical School; Ethics Commission of the Christian-Albrechts-University Kiel; Ethics Committee at the University of Würzburg Medical School; Ärztekammer des Saarlandes; Ethics Committee Heidelberg University) approved the studies. An overview of all patients is presented in Additional file 1: Table S1. Selected diseases/traits have been grouped together as “others”. This includes patients with unclear diagnosis, e.g., patients that have either a pancreatic cancer or pancreatitis or patients with prostate cancer or benign prostate hyperplasia. The “others” group also contains some very small cohorts, e.g., 6 samples with “atopic dermatitis”. One group has been left out of the pairwise comparisons, namely the 15 long-lived individuals that show a substantial age bias since these would potentially bias either the control or disease profiles.

### miRNA extraction and microarray screening

miRNA extraction and microarray measurement have been carried out as previously described [29]. The full data set has been deposited in the gene expression omnibus under reference GSE61741.

### Statistical analysis

All statistical computations were carried out using the publicly available statistical language R [30]. For each miRNA, we report median expression in the respective groups, together with fold changes. Beyond this information, the variability of miRNAs is of high importance. Thus, we calculated measures that also depend on the variance. To assess the information content of single miRNAs and miRNA profiles, the area under the receiver operator characteristics (ROC) curve (AUC) was computed using the pROC package. The 95% confidence intervals for the ROC curves and AUC values were calculated using 2,000 bootstrap samples. To determine significance values for miRNAs, two-tailed unpaired *t*-tests were calculated and the significance values adjusted for multiple testing using the Benjamini-Hochberg approach. Validated target miRNAs by reporter assays have been extracted from miRTarBase [31,32]. Pathway enrichment analysis has been carried out using our tool GeneTrail [33,34]. Visualization has been done using CytoScape.

### Machine learning analysis

Supervised classification of samples was carried out using linear Support Vector Machines (SVM) as implemented in the R e1071 package. SVMs were evaluated by applying standard 10-fold cross-validation and a stepwise-forward filter subset selection technique. In order to account for variations in the random partitioning into sample subsets, cross-validation runs were repeated 10 times. Moreover, to test for potential overtraining, exactly the same procedure was carried out using randomly permuted class labels, such that 10 so-called permutation tests were applied for each subset size. All classifications were carried out with equal cohort sizes, i.e., if one group was larger than the other, samples from the first group were randomly selected in each repetition in order to simulate the same cohort sizes.

### qRT-PCR validation

qRT-PCR was performed in two participating centers (center 1: Heidelberg University, center 2: Saarland University) using the miScript PCR System (Qiagen) and the primer assays for hsa-miR-144* and hsa-miR-155*. We analyzed the expression of these two miRNAs in a total of 282 samples (center 1: 172 samples from controls, and patients with acute myocardial infarction, non-ischemic systolic heart failure, glioblastoma, pancreatic diseases, and breast cancer; center 2: 110 samples from controls, and patients with Wilms tumor, psoriasis, renal cancer, prostate cancer, lung cancer, multiple sclerosis, benign prostate hyperplasia, colon cancer, and chronic obstructive pulmonary disease). Additionally, a third cohort was included, providing 37 samples of a breast cancer study, a phenotype that was not included in the screening. As endogenous control, RNU6B was measured. To analyze qRT-PCR data we applied relative quantification using the 2^−ΔΔ*C*^_T_ method [35].

## Results

### Identification of miRNAs generally indicating the presence of a disease

**Figure 1:**
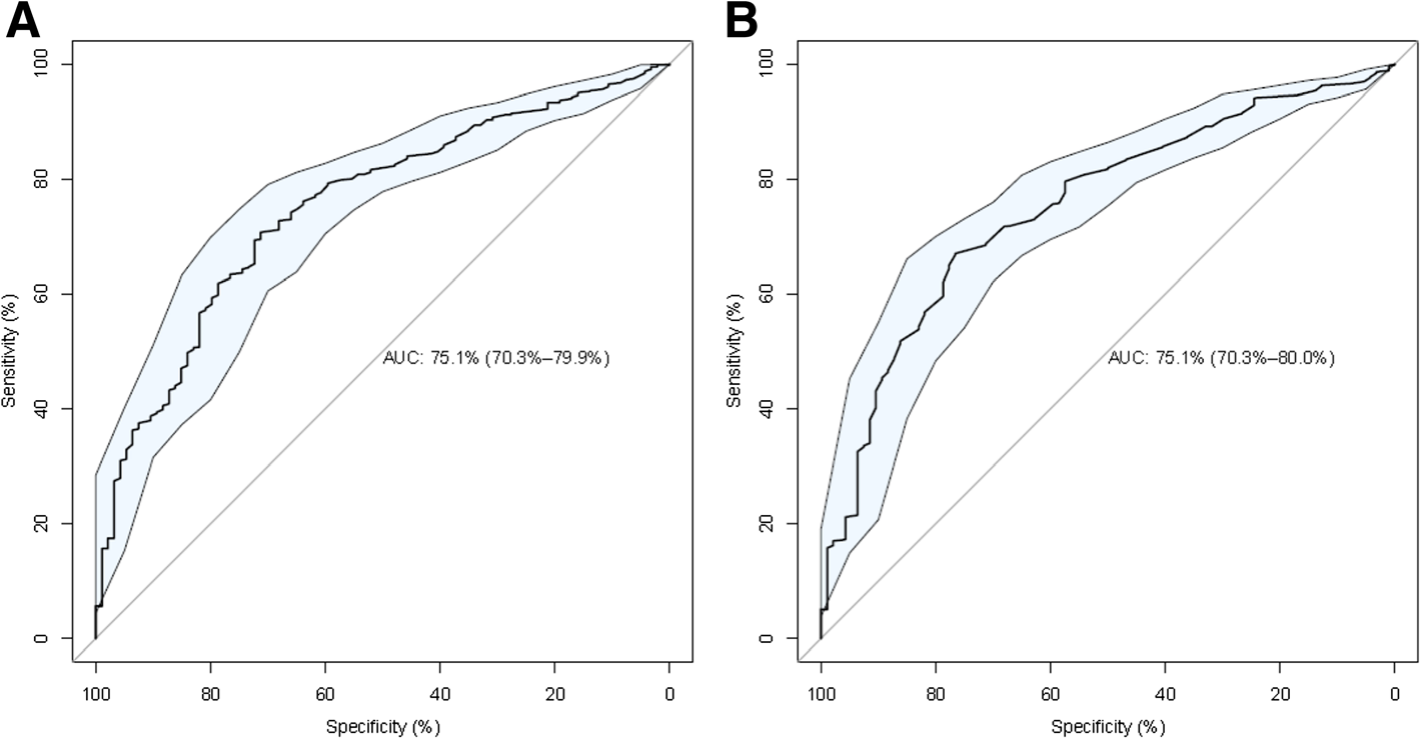
**ROC curves for disease specific miRNAs. (A)** The ROC curve for hsa-miR-144* is shown. **(B)** The ROC curve for hsa-miR-20b is shown. The blue shaded area denotes the 95% confidence interval computed by 2,000 bootstrap samples.

We asked if there is a general association between the expression of certain miRNAs in peripheral blood and the presence of a disease. To this end, we calculated a two-tailed unpaired *t*-test of all patients versus all controls and adjusted the significance values for multiple testing. Furthermore, we calculated for each miRNA separately the AUC together with the respective 95% confidence intervals. For the comparison of diseases (cancer and non-cancer samples) versus healthy controls, we found 333 statistically significantly dysregulated miRNAs (adjusted *P* <0.05), of which 254 were upregulated in diseases while only 79 were downregulated. The most significant miRNA hsa-miR-576-5p reached an adjusted significance value of 4.7 × 10^−16^ (raw *P* =5.6 × 10^−19^). The miRNAs hsa-miR-144* and hsa-miR-20b were the most downregulated with an AUC of 0.751 (95% CI: 0.703–0.799), followed by miR-17 and miR-20a. For the first two miRNAs, ROC curves are presented in Figure 1. In contrast, hsa-miR-720 was the most upregulated with an AUC value of 0.68, followed by hsa-miR-302c. All AUC and *P* values for this comparison are provided in Additional file 1: Table S2. As this table demonstrates, some miRNAs, such as miR-576-5p, had a median expression close to the background. However, interpreting the actual expression values it can be seen that many patients partially demonstrate a very high expression of that miRNA. Here, the mean value of the samples may help to interpret the expression level and differences in miRNA abundance. For controls, the mean of this miRNA is 5.4, signifying an expression level close to the background. However, for patients, the mean is clearly above the background with a value of 21.4, i.e., four times higher as for controls. This may indicate that such generally low abundant miRNAs may have an influence on diseases.

Focusing on onco-miRNAs by comparing cancer samples versus healthy controls we found 322 miRNAs with adjusted *t*-test *P* <0.05, of which 101 were downregulated in cancer while 221 were upregulated. The most significantly dysregulated miRNA, hsa-miR-130b*, reached an adjusted significance value of 1.9 × 10^−14^ (raw *P* =2.2 × 10^−17^). In this analysis, again hsa-miR-144* and hsa-miR-20b showed the strongest downregulation in diseases with AUC values of 0.771 (95% CI of 0.721–0.821) and 0.760 (95% CI of 0.71–0.811), respectively, while hsa-miR-194* was the most upregulated miRNA with an AUC value of 0.687. All AUC and *P* values for this scenario are provided in Additional file 1: Table S3.

**Figure 2:**
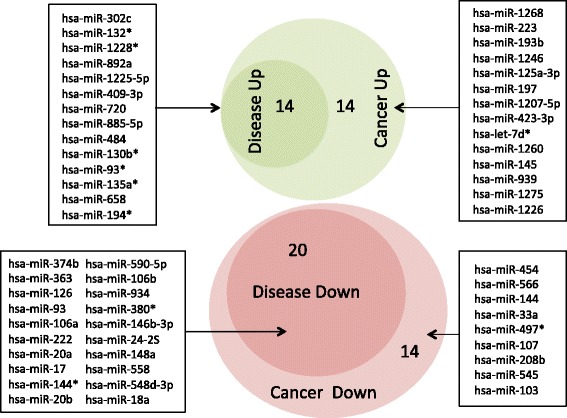
**Area-proportional Venn diagram for miRNAs with the highest AUC values in the comparisons of diseases versus healthy controls and cancer versus healthy controls.** Green area shows upregulated miRNAs while red area shows downregulated miRNAs in cancer and diseases in general. Both comparisons show a high overlap of dysregulated miRNAs, the respective miRNAs are presented on the left and right of the Venn diagram.

Notably, both comparisons described above showed a high concordance, demonstrated by a correlation of 0.95 of the AUC values and the significant overlap presented in the Venn diagram (Figure 2). This result indicates that most miRNAs are not specific for cancer but for diseases in general. Thus, it is not surprising that the maximal AUC between all cancer and non-cancer diseases computed for hsa-miR-574-5p was just 0.63 and is thus substantially smaller than the AUCs for the comparison of diseases versus healthy control samples. Likewise, we found a decreased number of miRNAs significant for this comparison. Altogether, just 116 miRNAs reached a significance value of below 0.05 and remained significant after adjustment for multiple testing. Of these, 61 were downregulated in cancer and 55 were upregulated. All AUC and *P* values for this comparison are provided in Additional file 1: Table S4.

### Disease-specificity of single miRNAs

**Figure 3:**
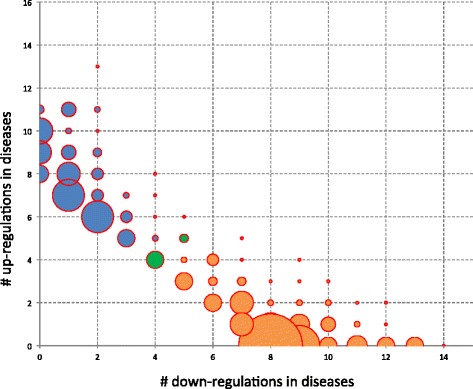
**Up- versus downregulations.** The balloon plot shows, for the different miRNAs, how many diseases the miRNAs are up- and respectively downregulated in. The bubble size represents the number of miRNAs showing this distribution in up- and downregulation. Orange bubbles belong to predominantly downregulated while blue bubbles belong to predominantly upregulated miRNAs. The two green bubbles represent 9 miRNAs that were equally up- and downregulated in disease.

As described above we found many miRNAs being dysregulated in diseases in general. To further explore this we asked how specific miRNAs are with respect to a specific disease, e.g., whether they are upregulated in one group and downregulated in another group of diseases. First, we compared all diseases separately against controls. Of all miRNAs, seven (hsa-miR-380*, hsa-miR-106b, hsa-miR-17, hsa-miR-144*, hsa-miR-558, hsa-miR-548d-3p, and hsa-miR-222) were significantly downregulated (adjusted two-tailed *t*-test *P* <0.05) in at least 13 of 19 disease conditions, representing the most non-specific miRNAs. A further 6 miRNAs were significantly downregulated in 12 pathologies, 7 miRNAs were downregulated in 11 pathologies, and 6 miRNAs were downregulated in 10 diseases while not being upregulated in any other. Conversely, three miRNAs (hsa-miR-130b*, hsa-miR-145, and hsa-miR-658) were upregulated in 11 diseases while not being downregulated in any other. Additionally, 9 miRNAs (hsa-miR-484, hsa-miR-499-5p, hsa-miR-126*, hsa-miR-491-5p, hsa-miR-1303, hsa-miR-539, hsa-miR-25*, hsa-let-7e*, and hsa-miR-194*) were upregulated in 10 diseases while not downregulated in any other, as the balloon plot (Figure 3) of all miRNAs significant in at least 8 of 19 diseases (>40%) shows. The balloon plot size represents the number of miRNAs that show significant up- and respectively downregulation in the calculated number of diseases. The largest bubble at position (8,0) represents 22 miRNAs that are downregulated in 8 diseases but not upregulated in a single disease. Altogether, 249 miRNAs are contained in the balloon plot. The respective markers can be found in Additional file 1: Table S5. Our results also provide strong evidence that up- and downregulation of miRNAs in diseases are anti-correlated, i.e., the dysregulated miRNAs are either up- or downregulated in diseases generally but very few miRNAs are upregulated in several diseases while downregulated in others. In our initial study [29], 62 miRNAs were found to be associated with over 40% of all tested disease conditions. Of these 62 miRNAs, 39 were found to be still dysregulated in at least 40% of all diseases despite our substantial extension of the study.

Importantly, we found a substantial variance in miRNA expression related to human pathologies. Considering single diseases, we found the highest number of 408 significantly dysregulated miRNAs in the case of colon cancer and melanoma. The lowest number with 115 dysregulated miRNAs was detected for pancreatitis. For each disease, we were furthermore able to detect a unique signature, i.e., a combination of significant miRNAs that did not overlapped with any other signature, allowing for specific differentiating between normal controls and diseases.

Besides the comparison between controls and diseases we also asked for specific signatures between diseases overall. Altogether, our study includes 20 different classes, 19 diseases as well as controls. Thus, a total of 190 specific signatures, one for each possible pair of the 20 cohorts, can be calculated. We carried out all comparisons and computed the number of miRNAs significant in each comparison as well as the number of comparisons where a certain miRNA was found to be significant. Thereby, we detected an average of 256 significant miRNAs per comparison. While some miRNAs were significant in many scenarios (including hsa-miR-106a (130 comparisons), hsa-miR-361-5p (130 comparisons), hsa-miR-17 (125 comparisons), hsa-miR-423-5p (125 comparisons), hsa-miR-320d (122 comparisons), and hsa-miR-20a (120 comparisons)), others were significantly dysregulated in just a few comparisons (including hsa-miR-506 (3 comparisons), hsa-miR-202* (5 comparisons), hsa-miR-361-3p (6 comparisons), hsa-miR-429 (7 comparisons), hsa-miR-548a-3p (9 comparisons), or hsa-miR-518e (9 comparisons)). All disease-specific signatures are detailed in Additional file 1: Table S5. In particular, the miRNAs that are significant in many different comparisons show a substantial data variance. To further evaluate this, we carried out an analysis of variance (ANOVA). Even after adjustment for multiple testing all but 19 miRNAs (2.2%) were significant in our ANOVA. The highest significance was reached for hsa-miR-151-3p (*P* = 4.03 × 10^−89^). Among the most significant miRNAs in the ANOVA was also hsa-miR-144*, being significant in 14 different diseases and representing the most generally dysregulated miRNA with a significance value of 1.88 × 10^−33^. Among the miRNAs with higher significance values, we found hsa-miR-155* to be significantly downregulated in just two diseases, namely acute myocardial infarction and glioma.

### qRT-PCR validation of microarray data

To validate our microarray results for two important disease miRNAs, hsa-miR-144* (non-specific) and hsa-miR-155* (specific), qRT-PCR was performed in two participating centers. Center 1 (Heidelberg University) analyzed a total of 172 samples from controls and patients with acute myocardial infarction, non-ischemic systolic heart failure, glioblastoma, and pancreatic diseases. Center 2 (Saarland University) analyzed a total of 110 samples from controls and patients with Wilms tumor, psoriasis, renal cancer, prostate cancer, lung cancer, multiple sclerosis, benign prostate hyperplasia, colon cancer, and chronic obstructive pulmonary disease samples.

For miR-144*, we measured ΔΔCT values of −1.93 in center 1. Thus, hsa-miR-144* was downregulated 3.8-fold in diseases (*P* =1.9 × 10^−5^). In center 2, we calculated ΔΔCT values of −1.75; thus, concordantly hsa-miR-144* was significantly less expressed in diseases (*P* = 0.0096) with a fold-change of 3.4.

As an independent set of patients and controls, we selected a third cohort of samples, containing blood samples from controls and from breast cancer patients. Notably, this validation was independent, in that the phenotype has not been included in the initial microarray screening and, likewise, this center had not contributed any samples to the initial screening (sample details are provided in Additional file 1: Table S6). The qRT-PCR was performed from center 1. The ΔΔCT value was −1.79. As for the first two validation approaches, hsa-miR-144* was significantly less (*P* =0.04) expressed with a fold-change of 3.5 in breast cancer samples compared to controls. In summary, we were able to successfully validate that hsa-miR-144* was significantly downregulated in various diseases in a total of 319 samples over three approaches with consistent fold-changes of 3.8, 3.4, and 3.5, respectively.

Analogously to hsa-miR-144* as a general disease marker, we also validated the miRNA hsa-miR-155* as example of a rather specific miRNA. In our microarray experiments hsa-miR-155* was only significantly downregulated in two diseases, namely acute myocardial infarction and glioma. The validation in center 1 reached a highly significant *P* value of 3.66 × 10^−6^, showing a significant downregulation of this miRNA in diseases (on average 2.8-fold). Remarkably, as mentioned above the sample cohort analyzed in this center contained acute myocardial infarction samples and glioma samples, as well as non-ischemic systolic heart failure and pancreatic diseases. However, in discordance with the screening results, we also found downregulation of miR-155* for pancreatic diseases. In the second validation in center 2, analyzing besides controls the diseases Wilms tumor, psoriasis, renal cancer, prostate cancer, lung cancer, multiple sclerosis, benign prostate hyperplasia, colon cancer, melanoma, and chronic obstructive pulmonary disease we found a slight upregulation of has-miR-155* at a moderate fold-change of 1.7 with a non-adjusted significance value of 0.008. After adjusting for multiple testing, only one of the 10 tested diseases (prostate cancer) remained significant. For the breast cancer samples against controls we likewise did not detect any statistically significant difference (*P* =0.42), providing evidence that hsa-miR-155* is in contrast to hsa-miR-144*, and is not a general disease marker but only significant in a restricted subset of diseases.

### Improvement of AUC values by combining multiple miRNAs

As demonstrated, miRNAs have the potential to differentiate between controls and patients in general with high AUC values up to 0.75. By combining the predictive power of different miRNAs it can be expected that the diagnostic power increases. To test this hypothesis we employed a machine learning procedure. We applied a stepwise forward subset selection approach with radial basis function SVM and carried out 10 random repetition of 10-fold cross-validation.

**Figure 4:**
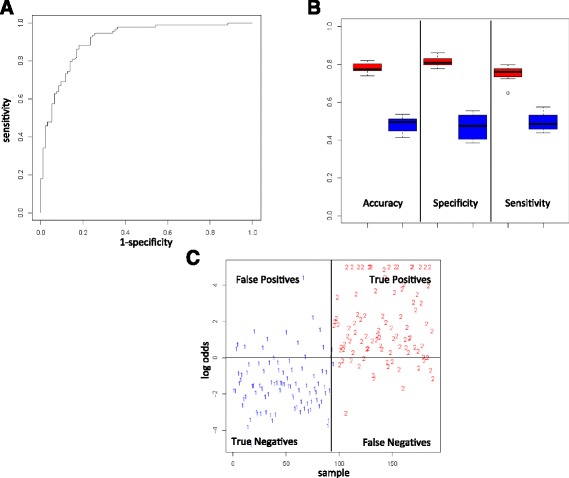
**Classification in patients (cancer and non-cancer) and controls. (A)** ROC curve for the best classification. **(B)** Box-plots for accuracy, specificity, and sensitivity for the 10 repeated cross validations in red and for 10 permutation tests in blue. **(C)** The best classification. Samples above the horizontal black line are considered as patients (denoted by 2) and below the black line as controls (denoted by 1).

For the classification in control and disease samples we reached maximal AUC values of 0.911, as the ROC curve in Figure 4A demonstrates. Our classifier outperformed the maximal AUC of the best single biomarker, i.e., hsa-miR-144* and hsa-miR-20a (AUC 0.751, respectively), by 16%. Altogether, we reached classification accuracy, specificity and sensitivity of 78%, 81%, and 75%, as the box-plot in Figure 4B details. These results are significantly improved as compared to random permutation tests, presented as blue boxes in Figure 4B (*P* <10^−10^). Figure 4C presents the classification example leading to the best AUC of 0.911, providing evidence that the majority of the samples have been classified correctly.

For the comparison of cancer versus controls the highest AUC was as high as 0.94, representing a 16.9% improvement over the best single miRNA for this comparison (hsa-miR-144*). Overall, a classification accuracy of 82%, a specificity of 81%, and a sensitivity of 83% were reached.

### Target analysis of dysregulated miRNAs

**Figure 5:**
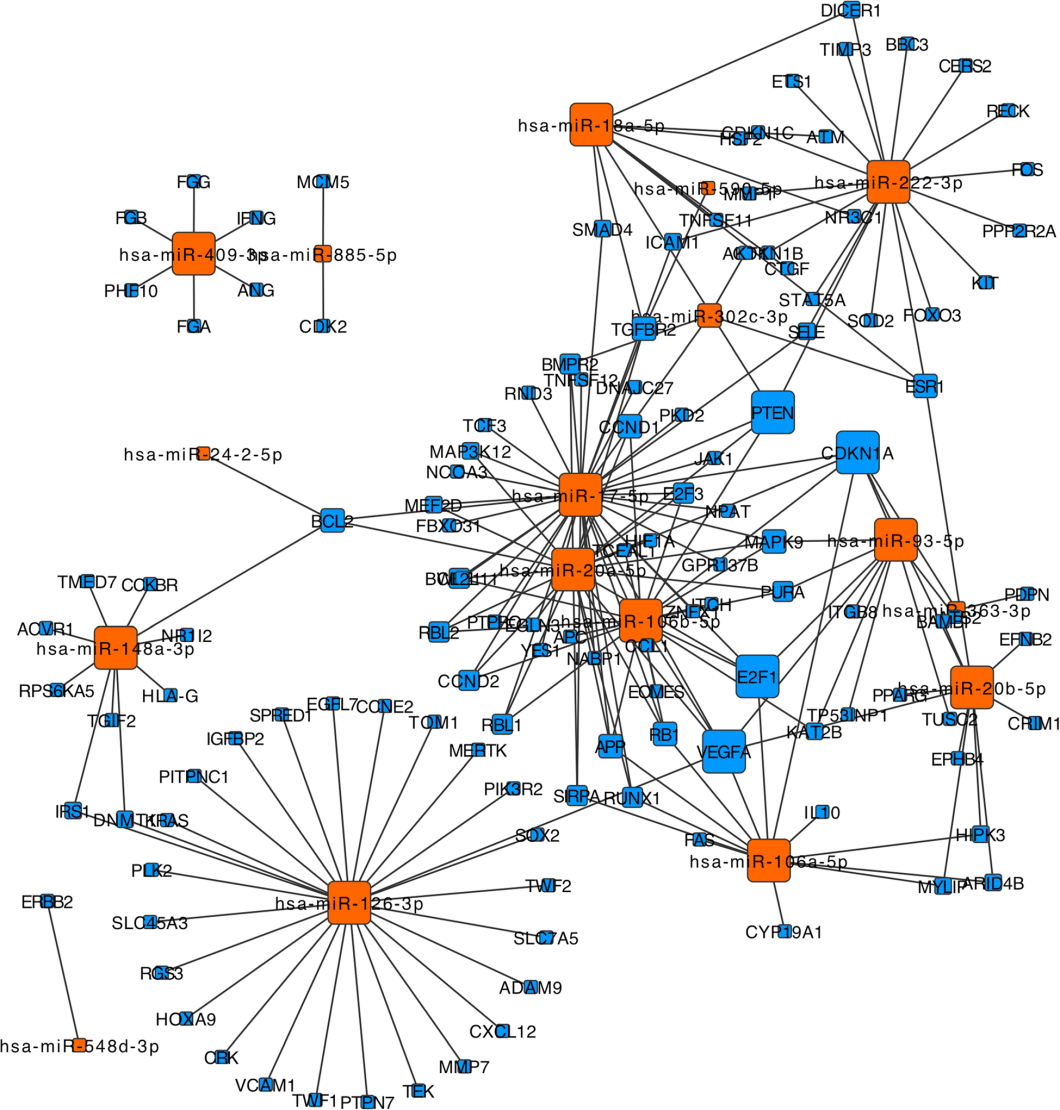
**miRNA-target gene network.** miRNAs are shown as orange nodes and target genes that have been detected by reporter assays as blue nodes. The node size corresponds to the degree of the respective nodes. In particular, the large blue nodes, i.e., genes that are regulated by many disease-related miRNAs, are of interest.

To gain insights into the molecular function of the miRNAs, we carried out a network analysis. First, we extracted all targets of the 34 miRNAs that are associated with diseases in general. Since *in silico* predictions may show many false positive interactions or miss identifying actual miRNA-target gene relations we considered only experimentally validated targets. Specifically, we considered miRNA-target gene associations that have been verified using reporter assays. The respective 199 interactions between the miRNAs and target genes have been extracted from the miRTarBase. The interaction graph is presented in Figure 5, showing miRNAs as orange nodes and target genes as blue nodes with the node sizes representing the degree (i.e., the number of neighbors) of the miRNAs and target genes. Of high interest are genes that are targeted by different miRNAs. Especially, CDKN1A, VEGFA, PTEN, and E2F1 were regulated by at least five miRNAs, VEGFA and CDKN1A even by seven different miRNAs. A further seven genes were regulated by four miRNAs: TGFBR2, RB1, CCND1, APP, BCL2, ESR1, MAPK9. To understand whether the regulated genes have a common biological meaning we carried out a network enrichment analysis using GeneTrail using the KEGG database [34]. We discovered significant associations with various different pathologies analyzed in our study. Most prominently, 32 target genes were related to pathways in cancer. Although these results do not demonstrate a direct relation between the miRNAs and the diseases on a functional level, the results indicate a potential key role of the disease-affected miRNAs in human pathogenic processes.

## Discussion

For most diseases, early and specific markers are lacking. Hence, besides the continuous refinement of existing biomarkers, the search for novel, early disease predictors belongs to the current challenges in biomarker research. miRNAs offer a new class of biologically active molecules that contribute to many disease processes and compensatory mechanisms. Accordingly, they might not only offer the ability to detect a disease early, but could also complement existing molecular and clinical markers by providing additional information, supporting a biomarker-guided differential diagnosis. Furthermore, miRNA signatures could support a differential diagnosis in clinically overlapping diseases, such as non-ischemic systolic heart failure versus acute myocardial infarction. Consequently, miRNAs are increasingly recognized as valuable biomarkers for different pathologies. However, in most studies, a case–control scenario has been applied and comprehensive comparisons between different diseases are largely missing.

The current meta-analysis aimed to compare the miRNA profiles from 1,049 samples belonging to 19 different diseases as well as controls. Here, we not only identified disease-specific miRNAs but also miRNAs associated with the presence of a disease in general. Moreover, we were able to show that miRNA patterns improve the diagnostic accuracy substantially and provide the required specificity for diagnostic purposes.

In the present study, we found many miRNAs that were either up- or downregulated in the majority of diseases compared to controls. Interestingly, among the most significant miRNAs downregulated in about 70% (13 of 19) of the analyzed diseases we found members of the miR-17 family, i.e., hsa-miR-17 and hsa-miR-106b. It is known that members of this family are over-expressed in cancer tissue and thus act as oncogenes by promoting cell proliferation, suppression of apoptosis of cancer cells, or induction of tumor angiogenesis [36]. Although this observation appears to be in contradiction to our data, one has to bear in mind that we analyzed blood but not tissue. The same holds for hsa-miR-144*, which was a key miRNA in our analysis and downregulated in almost all tested disease conditions. According to the Human MIRNA & Diseases Database (HMDD, [37,38]) several studies revealed hsa-miR-144* (in the current V20 miRBase: hsa-miR-144-5p) as disease-associated. In our recent study on Alzheimer’s disease, hsa-miR-144-5p was the most significantly downregulated miRNA in whole blood [28]. In addition, this miRNA was downregulated in esophageal biopsy specimens of eosinophilic esophagitis patients [39]. In contrast, Liu et al. showed that hsa-miR-144* is overexpressed in peripheral blood mononuclear cells of active tuberculosis patients [40] and Redova et al. showed that it is also upregulated in serum of patients with renal cell carcinoma compared to healthy controls [41]. hsa-miR-144* was further identified as a new fecal-based marker for colon cancer [42] and as significantly upregulated in primary medulloblastoma samples compared to neural stem cells [43]. In the abovementioned studies, hsa-miR-144* has been described to be upregulated in diseases. While it is known that blood- and tissue-based regulation do not necessarily correlate [44], likewise, blood-based patterns and serum-based patterns for the same disease can vary substantially. There may be different reasons for this observation. First, we did not include infectious diseases in our study while Liu et al. focused on tuberculosis patients. Second, different blood collection and measurement systems have been applied in both studies, potentially leading to a systemic bias complicating a comparison between the studies. These heterogeneous results underline the need for a high degree of standardization of blood collection, miRNA processing protocols, measurement, and bioinformatics. In addition, it is certainly advised to only relate miRNA data that have been obtained by comparable conditions.

Next, we would like to address the origin of the miRNAs that are generally up- or downregulated in diseases. In a previous study, we compared the expression of up-, down-, and not regulated miRNAs in CD14, CD15, CD19, CD3, and CD56 positive cells [45]. The miRNAs that are upregulated in diseases in the present study showed strongest expression in CD19 cells in our previous study. The downregulated miRNAs in diseases were predominantly expressed in CD14, CD15, and CD56 cells. This holds especially for hsa-miR-144*, which was mostly expressed in CD15 cells compared to the other cell types according to the results of our previous study. Although the aforementioned differences between up- and downregulated miRNAs in general were statistically non-significant (*P* >0.05), the results indicate that the miRNAs associated with diseases are expressed at varying levels in different blood cell types. Remarkably, the samples in our cell separation study have been collected in EDTA blood tubes since PAXgene tubes lead to a cell lysis. As mentioned in the previous paragraph, the differences in the blood collection protocols will impact the comparison between disease miRNAs and miRNAs expressed in different cell types.

The dataset used for our meta-analysis has been generated over three years and the samples have been collected at nine different institutions. An obvious confounding variable that also may limit the applicability of miRNAs in clinical routine is the storage of samples over time. To minimize this, we used PAXgene tubes containing RNA stabilizing agents, allowing for storage of samples between −20 to −70°C for up to 50 months. We additionally checked the storage of RNA samples over a period of up to four months at −20°C. After two months, we still reached correlation of 0.89, which is well in the range of the platform’s technical reproducibility for blood samples. Even after four months, we still reached a correlation of 0.865 (detail in Additional file 1: Figure S1). For serum samples, we were even able to show the stability of miRNA expression for much longer periods of time (up to three decades) [16]. The highly consistent and significant results obtained in our meta-analysis thus confirm the robustness of the approach.

Although these results support the idea of miRNAs as future diagnostic biomarkers, there are various aspects that have to be considered. While a strength of our study set-up is the parallel analysis of many human diseases, the cohort sizes for some of the diseases analyzed is rather small. Since a small cohort size may lead to an overestimation of the actual clinical performance for the respective disease, the identified signatures await confirmation by larger independent patient cohorts. Additionally, in prospective studies, one needs to investigate the outcome given clinical end-points associated with the different disease signatures.

To develop diagnostic tests, it is important to consider all information about the source of the miRNAs. The association with diseases in general is only one factor that needs to be taken into account when considering miRNAs as disease-specific biomarkers. Confounding biological factors, such as age or gender of patients, as well as technical factors, such as storage conditions and processing protocols, are also essential in order to judge the value of miRNAs as biomarkers [46].

Another potential reason which may delay or even hinder the translation into clinical routine is the measurement system; miRNAs are relatively stable molecules and their quantification can be achieved by different methodologies. As such, miRNA quantification by PCR-based approaches shows a very high dynamic range and allows for absolute quantification, thus enabling testing in clinical routine. Furthermore, techniques for measuring sets of miRNAs as qRT-PCR are relatively inexpensive, fast, and established in most clinical laboratories, enabling testing in clinical routine.

In summary, we present a substantial meta-analysis of high-throughput miRNA data from patients’ blood samples. Our study presents miRNAs that are dysregulated in almost all patients, such as miR-144*, which was also validated using qRT-PCR. However, the respective miRNAs do not have to be omitted as specific markers for single diseases. In combination with other miRNAs, these biomarkers can add substantial diagnostic information to disease-specific signatures. Moreover, we were able to present specific miRNA patterns for all diseases and for all inter-disease comparisons besides few cases such as the separation of pancreatitis from pancreatic cancer. Finally, we were able to report sets of miRNAs being dysregulated in specific diseases, further promoting the investigation of miRNAs from peripheral blood as clinically relevant information carriers.

## Conclusions

In this study, we performed a meta-analysis of 1,049 miRNA profiles measured from whole blood samples. We discovered miRNAs that seem to be generally associated with diseases, most importantly miR-144*. This miRNA was validated technically and in an independent cohort of breast cancer patients by qRT-PCR. We provided first evidence that specific miRNA patterns exist for all diseases. Additionally, we report a set of miRNAs that seem to be rather robust in the patient’s blood.

Our study underscores the potential of miRNA signatures for diseases. To translate respective biomarker sets into clinical practice, further validation studies on independent cohorts are however essential. Finally, it is crucial to follow strict standards in blood collection and measurement of miRNA profiles in order to minimize technical bias.

## Additional file

Additional file 1: Table S1. Information on the 1,049 samples included in the study, matching the information stored in the Gene Expression omnibus. **Table S2.** Average expression, *P* values, and AUC for the comparison of diseases versus controls. **Table S3.** Average expression, *P* values, and AUC for the comparison of cancer diseases versus controls. **Table S4.** Average expression, *P* values, and AUC for the comparison of cancer diseases versus non-cancer diseases. **Table S5.** AUC values and *P* values for all pair-wise comparisons. **Table S6.** Patient characteristics of the independent breast cancer cohort. **Figure S1.** Stability analysis of miRNAs over 2 months for three individuals.

## Abbreviations

AUC: area under the ROC curve
ROC: Receiver operator characteristics
SVM: Support vector machines
