# Supplementary material for: miRNAs can be generally associated with human pathologies as exemplified for miR-144*
Source: BMC Med. 2014 Dec 3;12:224. doi: 10.1186/s12916-014-0224-0 (PMC4268797; doi:10.1186/s12916-014-0224-0)
Supplement: Additional file 1: Table S1. — Information on the 1,049 samples included in the study, matching the information stored in the Gene Expression omnibus. Table S2. Average expression, P values, and AUC for the comparison of diseases versus controls. Table S3. Average expression, P values, and AUC for the comparison of cancer diseases versus controls. Table S4. Average expression, P values, and AUC for the comparison of cancer diseases versus non-cancer diseases. Table S5. AUC values and P values for all pair-wise comparisons. Table S6. Patient characteristics of the independent breast cancer cohort. Figure S1. Stability analysis of miRNAs over 2 months for three individuals. [file 12916_2014_224_MOESM1_ESM.zip › Supplemental Figures.pptx]

## Slide 1
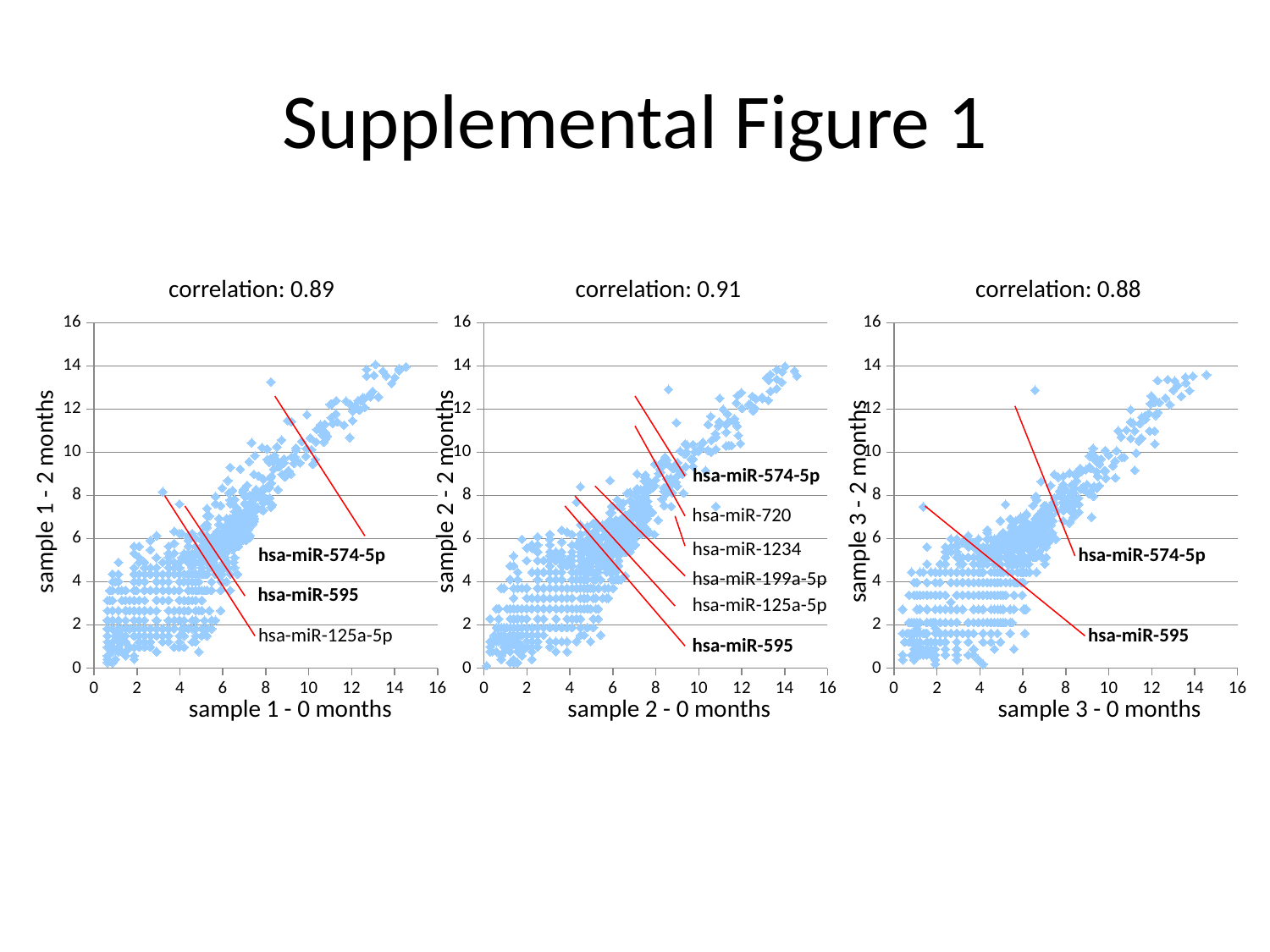

# Supplemental Figure 1
correlation: 0.89
correlation: 0.91
correlation: 0.88
### Chart
| Category | Sample03_2 |
|---|---|
### Chart
| Category | Sample04_2 |
|---|---|
### Chart
| Category | Sample05_2 |
|---|---|hsa-miR-574-5p
sample 1 - 2 months
sample 2 - 2 months
sample 3 - 2 months
hsa-miR-720
hsa-miR-1234
hsa-miR-574-5p
hsa-miR-574-5p
hsa-miR-199a-5p
hsa-miR-595
hsa-miR-125a-5p
hsa-miR-125a-5p
hsa-miR-595
hsa-miR-595
sample 1 - 0 months
sample 2 - 0 months
sample 3 - 0 months
